# Tick-Borne Relapsing Fever Caused by *Borrelia persica* in Traveler to Central Asia, 2019

**DOI:** 10.3201/2604.191771

**Published:** 2020-04

**Authors:** Veronika Muigg, Helena M.B. Seth-Smith, Daniel Goldenberger, Adrian Egli, Beatrice Nickel, Roland Dürig, Esther Kuenzli, Vladimira Hinic, Andreas Neumayr

**Affiliations:** Swiss Tropical and Public Health Institute, Basel, Switzerland; and University of Basel, Basel (V. Muigg, B. Nickel, E. Kuenzli, A. Neumayr);; University Hospital Basel, Basel (H.M.B. Seth-Smith, D. Goldenberger, A. Egli, V. Hinic);; University of Basel, Basel (H.M.B. Seth-Smith, A. Egli); Maihofpraxis, Luzern, Switzerland (R. Dürig)

**Keywords:** Tick-borne relapsing fever, Borrelia persica, central Asia, Borrelia species, shotgun metagenomics, microscopy, bacteria, vector-borne infections, Tajikistan, Switzerland

## Abstract

We report a case of tick-borne relapsing fever caused by *Borrelia persica* in a traveler returning to Switzerland from central Asia. After the disease was diagnosed by blood smear microscopy, the causative *Borrelia* species was confirmed by shotgun metagenomics sequencing. PCR and sequencing techniques provide highly sensitive diagnostic tools superior to microscopy.

We report a case of tick-borne relapsing fever (TBRF) in a 21-year-old male tourist who returned from Kyrgyzstan in July 2019 after having traveled for 5 months to Mexico, Taiwan, and central Asia (Uzbekistan, Tajikistan, and Kyrgyzstan). While in Tajikistan, he experienced acute fever of 39.5°C, chills, and generalized aches on June 11, which lasted 3 days. He experienced identical episodes around June 17 and 25. 

After returning to Switzerland, he sought care on June 28 from his general practitioner, who referred him to the regional hospital, where malaria test results were negative. After the patient experienced 2 more episodes of fever (July 2 and 14), the general practitioner referred him to a tropical medicine specialist on July 15. Anamnesis revealed that the patient had consumed unpasteurized milk and had been bitten by insects nightly while trekking in Tajikistan. Other than fever of 38.5°C and pain on palpation of the liver, physical examination revealed no pathologic findings. Abdominal sonography showed a borderline enlarged spleen but was otherwise unremarkable. Chest radiography indicated no abnormalities. Laboratory results are shown in the Appendix.

Detection of spirochetes in blood films (Figure) confirmed the diagnosis of a relapsing fever borreliosis, already suspected from the classical presentation of recurrent fever episodes separated by asymptomatic intervals of ≈1 week. Shortly after starting doxycycline, the patient experienced a self-limiting crisis with chills and fever of 41°C, which we interpret as Jarisch-Herxheimer reaction. Subsequently, the patient’s condition rapidly improved.

**Figure:**
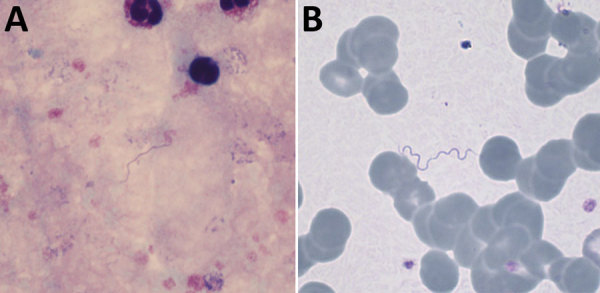
Giemsa-stained thick (A) and thin (B) blood films, demonstrating extracellular spirochetes. Original magnifications ×1,000.

To determine the *Borrelia* species, we performed 16S rRNA gene sequencing from the blood sample. Analysis of traces of capillary-sequenced amplified DNA after broad-range 16S rRNA gene PCR (660bp), performed by using RipSeqMixed (Pathogenomix, https://www.pathogenomix.com), could not differentiate between *Borrelia recurrentis* and *B. persica* within the 5′ end of the 16S rRNA gene. Therefore, we used a short-read shotgun metagenomic sequencing approach on DNA on an Illumina NextSeq500 platform (https://www.illumina.com).

 Of the 7.8 million sequencing reads, 692 (0.009% of the sequence data) mapped (by CLC Genomics Workbench v.12.0.3 [QIAGEN, https://www.qiagen.com] with a length fraction of 0.8 and a similarity fraction of 0.95) to a derived database of *Borrelia* genomes comprising reference genomes of *B. recurrentis* (GenBank accession nos. CP000999–CP001000), *B. persica* (Assembly accession AYOT), *B. duttonii* (Assembly accession AZIT), *B. hispanica* (Assembly accession AYOU), and *B. crocidurae* (GenBank accession no. LN609267). The top hit was to *B. persica*, with 684 (98.8%) mapped reads, followed by *B. duttoni* with 6 reads and *B. recurrentis* with 2 reads. Across the *B. persica* reference genome, reads from the isolate in this case mapped across the whole genome, representing sections of 101 of the 245 assembly scaffolds. We submitted the *Borrelia* reads to the European Nucleotide Archive (https://www.ebi.ac.uk/ena) under project PRJEB35490. We did not submit the 16S RNA gene sequence to GenBank because of the low quality of the sequence (multiple undetermined nucleotides). 

These results strongly suggest that *B. persica* was the infectious agent of TBRF. Pending microscopic confirmation, we ordered several serologic studies, including assays to detect antibodies against the *Borrelia* species that cause Lyme disease and against rickettsial pathogens (Appendix Table 1). Whether the mildly elevated serologic titer for spotted fever *Rickettsia* resulted from cross-reactivity or coinfection with a tick-borne *Rickettsia* remains unclear.

TBRF occurs in temperate and tropical countries and is caused by several species of *Borrelia* maintained in enzootic cycles in which small mammals serve as animal reservoirs and *Ornithodoros* soft ticks as vectors. Humans are accidental hosts (except for *B. duttonii* in Africa, which seems strictly limited to humans with no identified animal reservoir), usually exposed to tick bites when sleeping in rustic cabins or caves (*1*). The disease is characterized by recurrent fever episodes separated by afebrile periods and constitutional symptoms. Complications include meningoencephalitis and treatment-induced Jarisch-Herxheimer reaction. Diagnosis can be made by microscopic examination of blood smears collected during fever episodes or by molecular methods.

TBRF in international travelers is rare. The GeoSentinel Surveillance Network reported only 4 cases of relapsing fever among 24,920 returning febrile travelers during 1997–2006 (*2*), and we found only 40 other travel-related cases published since 1982 (Appendix Table 2). Most TBRF infections in travelers are caused by *B. crocidurae* and almost exclusively acquired in Senegal. Recently, a new species, *Candidatus* Borrelia kalaharica, was found in 2 travelers to southern Africa (*3*,*4*).

Reports on *B. persica* infections are few and largely restricted to Iran and Israel. Only 2 other cases of *B. persica* infections in travelers returning from Uzbekistan/Tajikistan have been reported (*5*,*6*). Considering the wide geographic distribution of the transmitting tick, *Ornithodoros tholozani* (India, Pakistan, Afghanistan, western China, Kazakhstan, Kyrgyzstan, Tajikistan, Turkmenistan, Uzbekistan, Iran, Iraq, Turkey, Cyprus, Syria, Jordan, Israel, Egypt, and Libya [*7**,**8*]), considerable underreporting and underrecognition is likely. Although apparently rare, central nervous system involvement and acute respiratory distress syndrome may complicate TBRF caused by *B. persica* (*9*).

For patients with periodic fever and supporting exposure risk, clinicians should consider a differential diagnosis of TBRF and carefully examine blood smears by microscopy. Increasingly available PCR and sequencing techniques provide highly sensitive diagnostic tools superior to microscopy.

Appendix Laboratory results for traveler with tick-borne relapsing fever caused by *Borrelia persica*, 2019, and list of reported cases of tick-borne relapsing fever in travelers.
