# Supplementary material for: Tick-Borne Relapsing Fever Caused by Borrelia persica in Traveler to Central Asia, 2019
Source: Emerg Infect Dis. 2020 Apr;26(4):824–6. doi: 10.3201/2604.191771 (PMC7101097; doi:10.3201/2604.191771)
Supplement: Appendix — Laboratory results for traveler with tick-borne relapsing fever caused by Borrelia persica, 2019, and list of reported cases of tick-borne relapsing fever in travelers. [file 19-1771-Techapp-s1.pdf]

Article DOI: https://doi.org/10.3201/eid2604.191771

Tick-Borne Relapsing Fever Caused by *Borrelia persica* in Traveler to Central Asia, 2019

Appendix

Appendix Table 1. Laboratory results for f the patient

Result		2019		
Laboratory parameter	Reference range	Jun 28	Jul 8	Jul 15
Hemoglobin (g/l)	140–175	114	126	120
Hematocrit (%)	36–48	34	36.9	36.7
Red blood cells (x 10^{12}/L)	4.5–5.9	3.9	4.3	4.3
Platelets (x 10^{9}/L)	150–400	160	199	229
Leukocyte (x 10^{9}/L)	4.5–11	6.5	4.9	6.8
Lymphocytes (%)	25–40			22.8
Neutrophils (%)	43–76			70.4
CRP (mg/L)	<5	44	23	165
ASAT (U/L)	<40			19.4
ASAT (U/L)	<41			43.6
gGT (U/L)	<66			69.2
Creatinine (µmol/L)	<97			107
Blood microscopy				
Plasmodia spp./Malaria		Negative		Negative
Spirochetes				Positive
Serologies				
Rickettsia, spotted fever group	1:64			1:128
Rickettsia, typhus group	1:64			<1:64
Orientia tsutsugamushi	1:80			<1:80
Coxiella burnetii phase 1 IgG				Negative
Coxiella burnetii phase 2 IgM				Negative
Coxiella burnetii phase 2 IgG (U/mL)	20–30			<20
Leptospira spp.	1:24			<1:24
Brucella spp. IgG				Negative
Borrelia spp. IgM (U/mL)	<18			173
Borrelia spp. IgG (U/mlL	<10			57
Borrelia spp. IgM Western blot:				Borderline:
OSpC (p23)				Negative
VlsE IgM				+++
BmpA (p39)				Negative
Borrelia spp. IgG Western blot:				Negative
VlsE				Negative
BmpA (p39)				Negative
p38				Negative
BBA36 (iv1)				Negative
BBO323 (iv2)				Negative
Crasp3 (iv3)				Negative
pG (iv4)				Negative

Appendix Table 2. Case reports on tick-borne relapsing fever in travelers

Year	No. cases	Infection acquired in	Imported to	*Borrelia* spp.	Complications	Ref.
1982	1	Namibia	South Africa	?*	None	(*1*)
1984	1	Senegal	Germany	?*	None	(*2*)
1985	1	Cyprus	England	?*	None	(*3*)
1988	1	Israel	USA	?*	Jarisch-Herxheimer reaction	(*4*)
1991	1	Senegal	Belgium	?*	Meningoencephalitis, Jarisch-Herxheimer reaction	(*5*)
1991	1	Senegal	Belgium	?*	None	(*5*)
1999	1	Senegal	Italy	?†	None	(*6*)
1999	2	Gambia or Senegal	Netherlands	*B. crocidurae*	Meningitis (n = 1)	(*7*)
2005	1	Morocco or Spain	France	*B. hispanica*	None	(*8*)
2005	1	Mali	France	*B. crocidurae*	None	(*8*)
2005	1	Senegal or Mauritania	France	*B. crocidurae*	None	(*8*)
2006	1	Senegal	Italy	*B. crocidurae*	None	(*9*)
2006	1	Guatemala or Belize	Netherlands	?†	None	(*10*)
2007	1	Mali	France	?†	None	(*11*)
2008	4	Senegal	France	?†[†]	Meningoencephalitis (n = 1) Jarisch-Herxheimer reaction (n = 1)	(*12*)
2008	1	Mali	France	?†	None	(*13*)
2009	1	Senegal	France	*B. crocidurae*	None	(*14*)
2010	1	Uzbekistan	Japan	*B. persica*	None	(*15*)
2010	1	Senegal	Belgium	*B. crocidurae*	Meningoencephalitis	(*16*)
2011	1	Uzbekistan or Tajikistan	France	*B. persica*	None	(*17*)
2009 - 2011	11	Senegal (n = 4), not stated (n = 7)	France	*B. crocidurae* (n = 11)	Meningitis (n = 4), Encephalitis (n = 2)	(*18*)
2012	1	Ethiopia	France	?‡	Radiculopathy	(*19*)
2015	1	Southern Africa	Germany	*Candidatus B. kalaharica*	None	(*20*)
2016	1	Southern Africa	Germany	*Candidatus B. kalaharica*	Jarisch-Herxheimer reaction	(*21*)
2017	1	USA	Japan	*B. miyamotoi*	None	(*22*)
2018	1	Senegal	France	?†	None	(*23*)

*Genotyping not yet available.
†No genotyping result reported.
‡Genotyping result inconclusive.

References

1. Rosenthal E. Relapsing fever in Cape Town. A case report. S Afr Med J. 1982;61:801–2. PubMed

2. Huber M, Eichenlaub D. Relapsing fever in a tourist to Africa [in German]. MMW Munch Med Wochenschr. 1984;126:178–80. PubMed

3. Simon JW. Tick borne relapsing fever imported into the United Kingdom. J R Army Med Corps. 1985;131:65–7. PubMed https://doi.org/10.1136/jramc-131-02-02

4. McNamara JJ, Kay HH. Relapsing fever (Borrelia) in an adolescent tourist in Israel. J Adolesc Health Care. 1988;9:421–3. PubMed https://doi.org/10.1016/0197-0070(88)90042-3

5. Colebunders R, De Serrano P, Van Gompel A, Wynants H, Blot K, Van den Enden E, et al. Imported relapsing fever in European tourists. Scand J Infect Dis. 1993;25:533–6. PubMed https://doi.org/10.3109/00365549309008539

6. Chatel G, Gulletta M, Matteelli A, Marangoni A, Signorini L, Oladeji O, et al. Short report: diagnosis of tick-borne relapsing fever by the quantitative buffy coat fluorescence method. Am J Trop Med Hyg. 1999;60:738–9. PubMed https://doi.org/10.4269/ajtmh.1999.60.738

7. van Dam AP, van Gool T, Wetsteyn JC, Dankert J. Tick-borne relapsing fever imported from West Africa: diagnosis by quantitative buffy coat analysis and in vitro culture of *Borrelia crocidurae*. J Clin Microbiol. 1999;37:2027–30. PubMed https://doi.org/10.1128/JCM.37.6.2027-2030.1999

8. Wyplosz B, Mihaila-Amrouche L, Baixench MT, Bigel ML, Berardi-Grassias L, Fontaine C, et al. Imported tickborne relapsing fever, France. Emerg Infect Dis. 2005;11:1801–3. PubMed https://doi.org/10.3201/eid1111.050616

9. Tordini G, Giaccherini R, Corbisiero R, Zanelli G. Relapsing fever in a traveller from Senegal: determination of Borrelia species using molecular methods. Trans R Soc Trop Med Hyg. 2006;100:992–4. PubMed https://doi.org/10.1016/j.trstmh.2005.11.002

10. Heerdink G, Petit PL, Hofwegen H, van Genderen PJ. A patient with fever following a visit to the tropics: tick-borne relapsing fever discovered in a thick blood smear preparation [in Dutch]. Ned Tijdschr Geneeskd. 2006;150:2386–9. PubMed

11. Gallien S, Sarfati C, Haas L, Lagrange-Xelot M, Molina JM. Borreliosis: a rare and alternative diagnosis in travellers' febrile illness. Travel Med Infect Dis. 2007;5:247–50. PubMed https://doi.org/10.1016/j.tmaid.2007.01.002

12. Patrat-Delon S, Drogoul AS, Le Ho H, Biziraguzenyuka J, Rabier V, Arvieux C, et al. Recurrent tick-borne fever: a possible diagnosis in patients returning from Senegal [in French]. Med Mal Infect. 2008;38:396–9. PubMed https://doi.org/10.1016/j.medmal.2008.03.005

13. Poirier P, Lebuisson A, Menager C, Moulin F, Dupouy-Camet J. Fever in a 7-year-old girl returning from Mali. Clin Infect Dis. 2008;47:1442, 1490–1. PubMed https://doi.org/10.1086/593096

14. Million M, Cazorla C, Doudier B, La Scola B, Parola P, Drancourt M, et al. Molecular identification of *Borrelia crocidurae* in a patient returning from Senegal. BMJ Case Rep. 2009;2009(feb19 1):bcr06.2008.0298. PubMed https://doi.org/10.1136/bcr.06.2008.0298

15. Kutsuna S, Kawabata H, Kasahara K, Takano A, Mikasa K. The first case of imported relapsing fever in Japan. Am J Trop Med Hyg. 2013;89:460–1. PubMed https://doi.org/10.4269/ajtmh.13-0187

16. Bottieau E, Verbruggen E, Aubry C, Socolovschi C, Vlieghe E. Meningoencephalitis complicating relapsing fever in traveler returning from Senegal. Emerg Infect Dis. 2012;18:697–8. PubMed https://doi.org/10.3201/eid1804.111771

17. Colin de Verdiere N, Hamane S, Assous MV, Sertour N, Ferquel E, Cornet M. Tickborne relapsing fever caused by *Borrelia persica*, Uzbekistan and Tajikistan. Emerg Infect Dis. 2011;17:1325–7. PubMed https://doi.org/10.3201/eid1707.101894

18. Goutier S, Ferquel E, Pinel C, Bosseray A, Hoen B, Couetdic G, et al. *Borrelia crocidurae* meningoencephalitis, West Africa. Emerg Infect Dis. 2013;19:301–4. https://doi.org/10.3201/eid1902.121325

19. Socolovschi C, Honnorat E, Consigny PH, Dougados J, Passeron A, Parola P, et al. Tick-borne relapsing fever with cutaneous eschar and radiculopathy, Ethiopia. J Travel Med. 2012;19:261–3. PubMed https://doi.org/10.1111/j.1708-8305.2012.00625.x

20. Stete K, Rieg S, Margos G, Häcker G, Wagner D, Kern WV, et al. Case report and genetic sequence analysis of Candidatus *Borrelia kalaharica*, southern Africa. Emerg Infect Dis. 2018;24:1659–64. PubMed https://doi.org/10.3201/eid2409.171381

21. Fingerle V, Pritsch M, Wächtler M, Margos G, Ruske S, Jung J, et al. "Candidatus *Borrelia kalaharica*" detected from a febrile traveller returning to Germany from vacation in southern Africa. PLoS Negl Trop Dis. 2016;10:e0004559. PubMed https://doi.org/10.1371/journal.pntd.0004559

22. Oda R, Kutsuna S, Sekikawa Y, Hongo I, Sato K, Ohnishi M, et al. The first case of imported *Borrelia miyamotoi* disease concurrent with Lyme disease. J Infect Chemother. 2017;23:333–5. PubMed https://doi.org/10.1016/j.jiac.2016.12.015

23. Guiheneuf E, Desjardins N, Guiheneuf R. A diagnosis of borreliosis on a blood smear [in French]. Ann Biol Clin (Paris). 2018;76:118–9. PubMed https://doi.org/10.1684/abc.2017.1320
